# miR-150-5p-Containing Extracellular Vesicles Are a New Immunoregulator That Favor the Progression of Lung Cancer in Hypoxic Microenvironments by Altering the Phenotype of NK Cells

**DOI:** 10.3390/cancers13246252

**Published:** 2021-12-13

**Authors:** Wei-An Chang, Ming-Ju Tsai, Jen-Yu Hung, Kuan-Li Wu, Ying-Ming Tsai, Yung-Chi Huang, Chao-Yuan Chang, Pei-Hsun Tsai, Ya-Ling Hsu

**Affiliations:** 1 Division of Pulmonary and Critical Care Medicine, Department of Internal Medicine, Kaohsiung Medical University Hospital, Kaohsiung Medical University, Kaohsiung 807, Taiwan; 960215KMUH@gmail.com (W.-A.C.); SiegfriedTsai@gmail.com (M.-J.T.); jenyuhung@gmail.com (J.-Y.H.); 980448kmuh@gmail.com (K.-L.W.); yingming@kmu.edu.tw (Y.-M.T.); 2 School of Medicine, College of Medicine, Kaohsiung Medical University, Kaohsiung 807, Taiwan; cyy807@gmail.com; 3 Department of Internal Medicine, Kaohsiung Municipal Ta-Tung Hospital, Kaohsiung Medical University, Kaohsiung 801, Taiwan; 4 Graduate Institute of Medicine, College of Medicine, Kaohsiung Medical University, Kaohsiung 807, Taiwan; beryl1992@gmail.com (Y.-C.H.); kanginbobo@gmail.com (P.-H.T.); 5 Department of Anatomy, Kaohsiung Medical University, Kaohsiung 807, Taiwan; 6 Drug Development and Value Creation Research Center, Kaohsiung Medical University, Kaohsiung 807, Taiwan

**Keywords:** CD226, extracellular vesicles, lung cancer, miR-150-5p, nature killer cell

## Abstract

**Simple Summary:**

Natural killer (NKs) cells are cytotoxic effector cells, which can modulate tumor metastasis according to their function. We determined the functional profiles of NK cells in a hypoxic tumor microenvironment (TME) of lung cancer, revealing CD226 downregulation and functional repression of NK cells after treated with hypoxic lung cancer-derived extracellular vesicles (EVs) containing miR-150-5p. We also found that NK cells from lung cancer patients had lower expression of CD226 on their surface and exhibited a pro-inflammatory, pro-angiogenic and tumorigenesis phenotype. Inhibition of miR-150 improved tumor surveillance by reversing CD226 expression and subsequently reinstating cytotoxic NK cell activity. Our study revealed a new scenario for the pro-inflammatory and pro-angiogenic activities of NK cells in the hypoxic TME in lung cancer.

**Abstract:**

Natural killer (NKs) cells are cytotoxic effector cells, which can modulate tumor metastasis according to their function; however, the role of NK cells in lung cancer has not been extensively investigated. In this study, we determined the functional profiles of NK cells in a hypoxic tumor microenvironment (TME) of lung cancer. We revealed CD226 downregulation and functional repression of NK cells after hypoxic lung cancer priming and we then investigated their interaction with extracellular vesicles (EVs) and miR-150-5p. We also found that NK cells from lung cancer patients had lower expression of CD226 on their surface and exhibited a pro-inflammatory, pro-angiogenic and tumorigenesis phenotype by expressing VEGF, CXCL1, CXCL8, S100A8 and MMPs. Moreover, inhibition of miR-150 improved tumor surveillance by reversing CD226 expression and subsequently reinstating cytotoxic NK cell activity in an animal model. Our study introduces a new scenario for the pro-inflammatory and pro-angiogenic activities of NK cells in the hypoxic TME in lung cancer.

## 1. Introduction

Lung cancer is the primary cause of cancer-related death worldwide [1]. Systemic treatment for lung cancer is continuously developing and includes options such as cytotoxic chemotherapy and targeted therapy. Immune checkpoint inhibition, including antibodies against cytotoxic T-lymphocyte-associated antigen 4 (CTLA-4), programmed cell death-1 (PD-1) and programmed cell death-ligand 1 (PD-L1), increases the therapeutic options available for metastatic lung cancer, and nowadays can be delivered as a monotherapy or a combination therapy; it also represents an alternative treatment opportunity for patients with advanced non-small cell lung cancer (NSCLC) after failure of target therapy [2,3]. Unfortunately, immunotherapy only benefits a small population of patients mainly because of two reasons: firstly, the mechanisms of immune escapade have not yet been effectively clinically targeted; secondly, the development of primary and acquired resistance [4,5]. This implies that there is a requirement to broaden the complexity of cross-talk between developing tumors and the host immune system.

The tumor microenvironment (TME) of a solid tumor is characterized by hypoxia, which is due to rapid cancer cell proliferation and metabolism, reduced nutrient transportation and low oxygen tension, and as a consequence of tumor angiogenesis, cancer progression and metastasis [6]. The TME displays inflammatory properties, which are orchestrated by the expression of cytokines/chemokines and their cognate receptors, by both tumor and stroma cells. This results in the recruitment of various immune populations, including tumor-associated macrophages, neutrophils, mast cells, and myeloid-derived suppressor cells (MDSCs), which interact with cancer cells and eventually shape an immunosuppressive environment, with diminished anti-cancer abilities and enhanced tumor-promoting manifestations.

Natural killer (NK) cells are the most crucial cytotoxic innate lymphocytes, which kill abnormal cells, such as cancer cells and cells infected by viruses [7]. The activation of NK cells is carefully controlled by a balance of activating and inhibitory receptors, including natural cytotoxicity receptors (NCRs) and killer cell lectin-like receptor (NKG2) molecules, NKG2D and NKG2A. Downregulation of activating receptor expression or upregulation of the inhibitory receptors on NK cells, is a strategy employed by tumors for immune evasion, and has been associated with tumor progression. CD226 (also known as DNAX Accessory Molecule-1, DNAM-1, PTA-1, and TLiSA1) is a surface receptor that is expressed on some types of immune cells, such as T cells, NK cells, B cells and monocytes, which has been shown to be required for cancer immunosurveillance [8]. The upregulated expression of NKG2A and downregulated level of CD226 were observed in the lung cancer group compared with the control group in a previous study [9]. Recent studies have shown the promotive role of NK cells in various cancers, including prostate cancer, colon cancer and NSCLC [10,11]. However, current research on various aspects of the pathophysiological role of NK cells in CD226 regulation has not yet been reported. The manipulation of NK function via CD226 expression may be a feasible strategy for the treatment of lung cancer.

Extracellular vesicles (EVs) have received increased attention in recent decades as critical mediators of intercellular interactions that contribute to various physiological and pathological processes [12,13]. EVs are lipid bilayers that transport a wide range of biologically active molecules, including proteins, lipids and non-coding RNAs, from host cells to recipient cells, thereby orchestrating phenotypic and functional alterations in the recipient cells [14,15]. Our study demonstrates that hypoxic lung cancer-secreted EVs containing miR-150 specifically changed the phenotypes of NK cells, which subsequently increased angiogenesis and immune suppression in the TME. Specifically, we show that miR-150 in lung cancer-secreted EVs contributes to the aforementioned processes by: (1) reducing the expression of CD226 on NK cells; (2) creating a favorable immunosuppressive microenvironment by releasing IL-10 and S100A8 protein; and (3) exploiting angiogenesis in the TME via VEGF, CXCL1 and CXCL8 production. Our study reveals that hypoxic lung cancer-secreted EVs impair NK cell function and could potentially be a therapeutic target for lung cancer.

## 2. Materials and Methods

### 2.1. Cell Culture and EV Isolation

Human adenocarcinoma cell line A549, NK cell line NK92 and human embryonic kidney cell line HEK-293, were purchased from American Type Culture Collection (Manassas, VA, USA), while the lung adenocarcinoma cell line CL1–5 cells were kindly provided by Prof. Pan-Chyr Yang in National Taiwan University. NK92 and CL1-5 cells were maintained at 37 °C, in a 5% CO_2_ humidified atmosphere, in complete RPMI1640 medium (Lonza, Basel, Switzerland) supplemented with 10% fetal bovine serum (FBS), 100  U/mL penicillin and 100  μg/mL streptomycin (Invitrogen, Carlsbad, CA, USA). HEK-293 cells were cultured in Eagle’s Minimum Essential Medium with 10% FBS, 100  U/mL penicillin and 100  μg/mL streptomycin (Invitrogen, Carlsbad, CA, USA). Human umbilical vein endothelial cells (HUVECs, Lonza, Basel, Switzerland) were cultured in endothelial cell basal medium (EBM, Lonza, Basel, Switzerland) supplemented with endothelial cell growth medium (EGM™ SingleQuots™, Lonza, Basel, Switzerland). HUVECs were used between three and six passages. Lung cancer cells were cultured in 1% oxygen (hypoxic condition) or 20% oxygen (normoxic condition) in exosome-depleted medium (Invitrogen, Carlsbad, CA, USA) for 24 h.

EVs were isolated from the culture medium of normoxic and hypoxic lung cancer cells as well as sera using exosome isolation kits (Invitrogen) after removing cell debris with centrifugation (1500 rpm for 10 min) as presented in our previous article [16,17,18]. The EVs were dissolved in PBS and stored at −80 °C. Purified EVs were identified by immunoblot for CD63, CD81, CD9 and HSP70.

### 2.2. NK Cell Isolation and Polarization

Peripheral blood mononuclear cells (PBMCs) from 10 different donors were isolated by centrifugation over Ficoll gradient (GE Healthcare, Freiburg, Germany). Primary NK cells were isolated from PBMCs with MACS technology using an NK Cell Isolation kit (Miltenyi Biotec, Cologne, Germany). To assess NK cell polarization, primary NK cells and NK92 cells were treated with EVs isolated from CL1-5 cells that had been incubated for 24 h under either normoxic or hypoxic (1% O_2_) condition (NK cells: CL1-5 cells = 1:10) in IL-2 (20 ng/mL) (R&D Systems, Minneapolis, MN, USA) containing RMPI 1640 for 5 days. After washing, conditioned media (CM) from the EV-treated NK cells were collected after another 48 h culture. We used a fixed ratio of secreted cells/recipient cells in order to reflect the parent cell status in the physiological condition, as adopted in many previous studies [16,17,19,20,21,22,23,24].

### 2.3. Flow Cytometry Analysis

To analyze surface markers, NK cells were stained with primary antibodies against a specific antigen or the corresponding isotype control antibodies, in PBS containing 2% (*w*/*v*) bovine serum albumin. The following antibodies were used: FTIC Mouse Anti-Human CD69 (Cat. no. 555530), APC Mouse anti-Human CD335 (NKp46) (Cat. no. 558051), PE Mouse Anti-Human CD226 (Cat. no. 559789), FITC Mouse Anti-Human CD16 (Cat. no. 555406), FITC Mouse Anti-Human CD56 (Cat. no. 562794), PE Mouse Anti-Human Granzyme B (Cat. no. 561142), FITC Mouse IgG1, κ Isotype Control (Cat. no. 555748), PE Mouse IgG1, κ Isotype Control (Cat. no. 555749) and APC Mouse IgG1, κ Isotype Control (Cat. no. 555751). All primary and isotype antibodies were obtained from BD Biosciences (San Jose, CA, USA). Flow cytometry data was acquired by Accuri C6 flow cytometer (BD Biosciences) and analyzed using CellQuest software (BD Biosciences).

Cell proliferation of NK cells induced by normoxic or hypoxic CL1-5-derived EVs plus IL-2 was assessed after labeling with 5-carboxyfluorescein diacetate succinimide ester (CFSE) for 5 days.

### 2.4. Functional Analysis of NK Cells

NK cell cytotoxicity against tumor cells (A549) was analyzed using a lactate dehydrogenase (LDH) release assay. Cells (2500 to 5000) were seeded in a 96-well plate and NK cells were then added at various ratios (1:1, 1:5, and 1:15, target cells: effector cells) the next day. After 4 h of co-culture, an aliquot of 50 μL media was used in the LDH cytotoxic assay kit (EMD Millipore, Billerica, MA, USA). The experimental release was corrected by subtraction of the spontaneous release of effector cells at the corresponding dilutions. % cytotoxicity = (experimental value − NK cell spontaneous control − target cell spontaneous control)/(target cell maximum control − target cell spontaneous control) × 100.

### 2.5. microRNA (miRNA)-Sequencing and Quantitative Real-Time PCR (qRT-PCR)

The miRNA and RNA profiles were examined using next-generation sequencing (NGS) as in our previous studies [25,26,27]. In brief, total RNA was extracted using TRIzol^®^ Reagent (Thermo Fisher Scientific, Waltham, MA, USA) following the instruction manual. The purified RNAs were quantified at OD_260 nm_ using a ND-1000 spectrophotometer (Nanodrop Technology, Wilmington, DE, USA) and qualitatively assessed using a Bioanalyzer 2100 (Agilent Technology, Santa Clara, CA, USA) with RNA 6000 LabChip kit (Agilent Technologies, USA). The quality of RNA should pass several pre-set criteria (OD_260_/OD_280_ = 1.8–2.0; OD_260_/OD_230_ ≥ 1.5; RNA integrity number [RIN] ≥ 8) before further processing. Library preparation, deep sequencing, trimming, alignment, and differential expression analyses were carried out by Welgene Biotechnology Company (Taipei, Taiwan) according to the Illumina’s official protocol and their well-developed pipeline.

miRNAs were reverse transcribed using the Mir-X™ miRNA First Strand Synthesis kit (Clontec). qRT-PCR was performed using SYBR Green Master Mix (Thermo Fisher Scientific, Inc., USA) with the QuantStudio 3 Real-Time PCR System (Applied Biosystems, ThermoFisher Scientific, Waltham, MA, USA). All reactions were performed in triplicate. U6 snRNA was used as the housekeeping gene and the relative standard method (2^−ΔΔCt^) was used to determine the relative miRNA expression. EV miRNA which was purified from serum was normalized with cel-miR-39 (Exiqon, Vedbaek, Denmark) as a spike-in control, and compared with the reference sample. Primer sequence for miR-150 was TCTCCCAACCCTTGTACCAGT. Primer sequences for pri-/pre-miR-150 were 5′-GTCCCCAGGTCCCTGTCC-3′ (forward) and 5′-CTGTCTCCCAACCCTTGTACC-3′ (reverse).

### 2.6. miRNA Mimics and Inhibitor Transfection, 3′UTR Luciferase Reporter Analysis

NK92 cells were transfected with control mimics, miR-150 mimic (100 nM), control inhibitor or miR-150 inhibitor (100 nM) using Dharmafect reagents (number 3) (Dharmacon, Lafayette, CO, USA). Oligonucleotides with random sequences served as negative controls for the miRNA mimic or inhibitor (Dharmacon).

HEK-293 cells were transfected with 0.4 μg of the luciferase reporter plasmid containing the predicted wild type miR-150-binding sites or mutated miR-150-binding sites/Renilla luciferase plasmid (Promega, Madison, WI, USA) (10:1), together with control mimics or miR-150 mimics. This was performed using DharmaFECT duo transfection reagent (Dharmacon) according to the manufacturer’s protocol. The cells were washed and luciferase activity was determined by the Dual-Luciferase Reporter Assay System (Promega). The relative luciferase activity was first normalized by the *Renilla* luciferase activity, then compared with the respective controls.

### 2.7. Network Formation Assay on Endothelial Cells

HUVECs (8 × 10^4^ cells/well) were seeded onto growth factor-reduced BD Matrigel (1 mg/mL) (BD Biosciences) in a 48-well plate. Cells were then supplemented for the condition medium (CM) of NK cells, which were treated with normoxic CL1-5 or hypoxic CL1-5-derived EVs for 5 days. After 8 h, HUVECs were stained with Calcein AM (Invitrogen, Waltham, MA, USA) for 30 min and imaged using an inverted Nikon Eclipse TE300 microscope (Nikon, Tokyo, Japan). 

### 2.8. Migration and Invasion Analysis

Cell migration or invasion assays were conducted using the 8-μm inserts (EMD Millipore) coated with (for invasion assay) or without (for migration assay) Matrigel. CL1-5 cells (1 × 10^5^ cells/well) were seeded in 8-μm pore inserts and CM of NK cells was added into the bottom wells for 24 h (for migration assay) or 48 h (for invasion assay) as chemoattractant. After staining with crystal violet, the migratory or invade cells were counted using a fluorescence microscope (Nikon’s ECLIPSE TE200 Inverted Microscope). 

### 2.9. Western Blot

The cells were lysed in RIPA lysis buffer (Millipore Corporation, Billerica, MA, USA), and equal amounts of protein were separated on sodium dodecyl sulfate–polyacrylamide gels. Anti-N-Cadherin (Cat. no. 610921), E-cadherin (Cat. no. 610182) and vimentin (Cat. no. 550513) antibodies were pursed from Becton Dickinson biosciences. Anti-actin, α-smooth muscle antibody (Cat. no. A5228) and GAPDH (Catalog# MAB374) antibodies pursed from EMD Millipore.

### 2.10. Quantification and Depletion of Cytokines

The various cytokine levels were determined by Luminex assays (R&D Systems, Minneapolis, MN, USA).

Cytokines depletion from the condition medium of various NK cells was performed by using anti-VEGF, anti-CXCL1, CXCL8 antibodies or control IgG (10 μg/mL, Millipore, Billerica, MA, USA) and Sepharose A/G beads following regular immunoprecipitation techniques. Immune complexes conjugated to protein A/G-Sepharose beads were precipitated by centrifugation. Cytokine depletion was confirmed by Luminex assays.

### 2.11. Fluorescent Imaging of EVs Uptake

EVs derived from CL1-5 cells were labeled with PKH-26 (Sigma–Aldrich, Burlington, MA, USA), and NK cells were stained with PKH-67 (Sigma–Aldrich, Burlington, MA, USA) for 30 min. After incubation with PKH-26-labelled EVs for 6 h, the NK cells were washed and then photographed using a Nikon inverted fluorescence microscope (ECLIPSE TE200 microscope).

### 2.12. In Vivo Delivery of miR-150-5p Inhibitor

Lewis lung carcinoma (LLC) (ATCC, CRL-1642) (1 × 10^6^), either miR-150 expressed or control plasmid transfected, were transplanted into mice (male, 8-week-old C57BL/6, *n* = 6) by tail vein injection. Animals were sacrificed on the 21st day and the number of tumor nodules in the lungs was counted. The anticancer effect of miR-150-5p inhibitor was assessed. Mice (male, 8-week-old C57BL/6, *n* = 6) were injected with LLC (1 × 10^6^) cells from the tail vein and were randomized to receive different treatments. Starting from the day after tumor cell injection, 5 μg of either control or miR-150 inhibitor mixed with 1.6 μL in vivo-JetPEI transfection reagent in 50 μL normal saline, was delivered to each mouse 7 times intravenously at a 3-day interval. The animals were sacrificed on the 21st day and the number of tumor nodules in the lungs were counted. The NK cells were isolated from the lungs of mice using NK cell isolation kits and the expression of CD226 (Cat. no. 565549, BD Biosciences.) was assessed by flow cytometry. All of the animal experiments were performed in accordance with the Institutional Animal Care and Use Committee guidelines, and with the approval of the Animal Care and Use Committee of the School of Kaohsiung Medical University.

### 2.13. Clinical Samples

Blood was collected from lung cancer patients and normal healthy subjects for isolation of primary NK cells and EVs. Approval for the study was obtained from the Institutional Review Board of Kaohsiung Medical University Hospital (KMUHIRB-E(I)-20180326). Written informed consent was obtained from all participants in accordance with the Declaration of Helsinki.

### 2.14. Statistical Analysis

Data are expressed as the mean ± standard deviation (SD) or standard error of the mean (SEM). Statistical differences between two groups were determined using Student’s t-test or Mann–Whitney test. For multiple comparisons, one-way analysis of variance (ANOVA) followed by Dunnett’s or Tukey’s post-hoc test was used. *p* values < 0.05 were considered statistically significant. All data were analyzed using the GraphPad Prism 9.2 (San Diego, CA, USA).

## 3. Results

### 3.1. NK Cells Treated with Hypoxic Lung Cancer Cell-Derived EVs Have Reduced Cytotoxic Function

**Figure 1:**
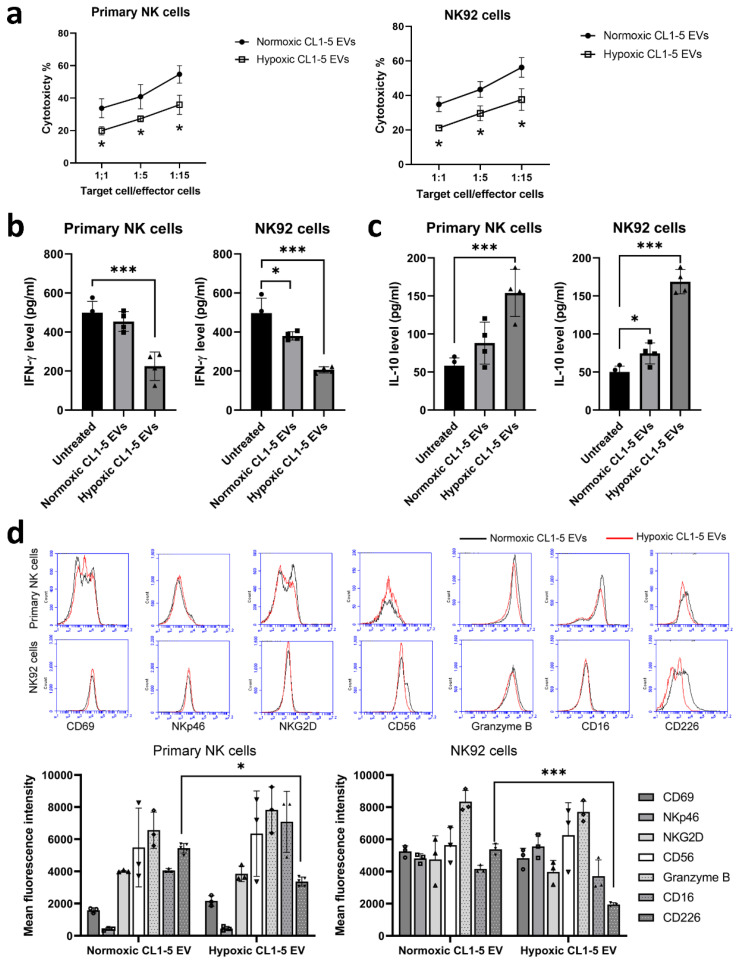
Hypoxic CL1-5-derived EVs (**a**) decreased NK cell cytotoxicity, (**b**) decreased INF-γ production and (**c**) increased IL-10 secretion. (**d**) The downregulation of CD226 expression in NK cells was caused by hypoxic CL1-5-derived EVs. Primary NK and NK92 cells were stimulated by EVs isolated from CL1-5 cells that had been incubated for 24 h under either normoxic or hypoxic (1% O_2_) condition at a 1:10 (recipient/secretory cells) ratio in IL-2-containing RMPI 1640 medium for 5 days. The expression of cytokines and surface markers was measured by a Luminex system and flow cytometry. Cell-mediated cytolysis was assayed using IL-2-stimulated primary NK and NK92 cells treated with normoxic or hypoxic CL1-5-derived EVs for 5 days. Target cells (A549) were included as an additional control. All data represent the median values of triplicate samples and are representative of 3 independent experiments. Error bars represent SDs. * *p* < 0.05, *** *p* < 0.005.

**Figure A1:**
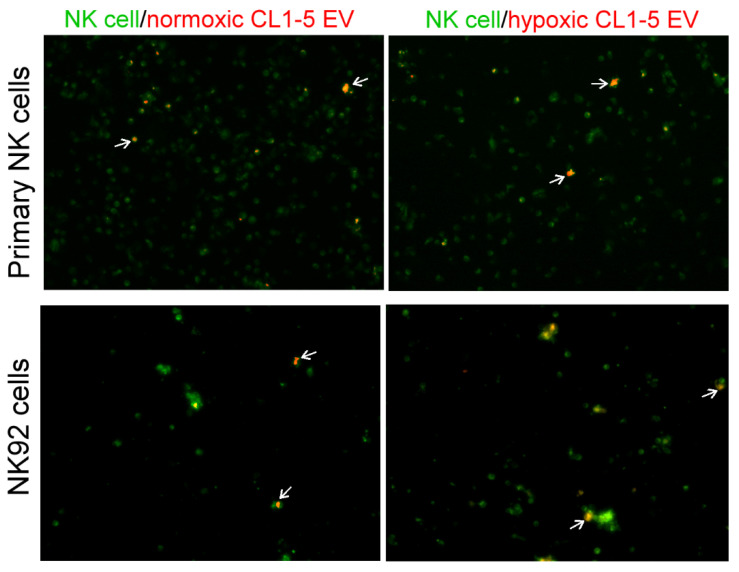
The uptake of CL1-5 cell-derived EVs in primary NKs and NK92 cells. PKH67 (**green**) staining NK cells were treated with PKH26-labeled EVs (**red**) for 6 h. The uptake of EVs in NK cells (orange cells, representatives are labelled with arrows) was captured by fluorescence microscopy.

To assess the role of hypoxia on cancer-mediated NK cell editing, we treated NK cells with EVs isolated from lung cancer CL1-5 cells that had been incubated for 24 h under either normoxic or hypoxic condition (1% O_2_). The uptake of lung cancer-derived EVs was observed by a fluorescence microscope (Figure A1 in Appendix A). The functional capacity of NK cell populations was determined by assessing their relative cytotoxic activity and cytokine levels. The lytic activity of NK cells was assessed on A549 target cells, showing that the cytotoxicity of hypoxic CL1-5 EV-treated primary NK and NK92 cells was markedly reduced compared with those treated with normoxic CL1-5 EVs (Figure 1a). Interestingly, decreased IFN-γ production and increased IL-10 production were observed in primary NK and NK92 cells treated with hypoxic CL1-5 EVs (Figure 1b,c). As the function of NK cells is regulated by a balance between signals, which are delivered by activating and inhibitory receptors, the phenotypes of primary NK and NK92 cells treated with EVs isolated from lung cancer cells under either normoxic or hypoxic conditions were determined. Flow cytometry showed that hypoxic lung cancer cell-derived EVs decreased the expression of CD226 on NK cells (Figure 1d), suggesting that hypoxic CL1-5-derived EVs downregulated the activating receptor CD226 and thereby impair the cytotoxicity of NK cells.

### 3.2. miR-150-5p in Hypoxic CL1-5-Derived EVs Influences NK Cell Function by Targeting CD226

**Figure 2:**
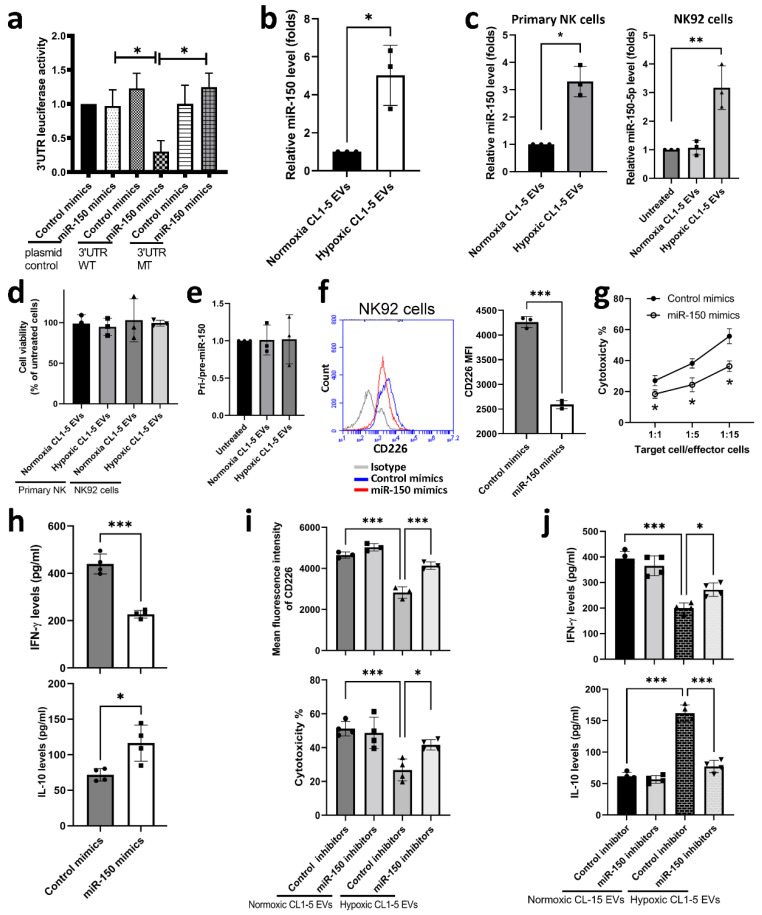
miR-150-5p was involved in downregulation of CD226 in NK cells. (**a**) The binding of miR-150-5p to the 3′UTR of *CD226* (wild type [WT] or mutated type [MT]) was determined by 3′UTR luciferase activity analysis. Increased miR-150-5p level was shown in hypoxic CL1-5 EVs (**b**) and primary NK and NK92 cells treated with hypoxic CL1-5-derived EVs (**c**). Treatment with CL1-5-derived EVs did not significantly alter the viability of primary NK and NK92 cells (**d**) or the expression of pri-/pre-miR-150 in NK92 cells (**e**). Transfection of miR-150-5p mimics decreased the expression of *CD226* (**f**), decreased cytotoxicity (**g**), decreased IFN-γ production (**h**), and increased IL-10 production (**h**) in NK92 cells. miR-150-5p inhibitors prevented the effects of hypoxic CL1-5 derived EVs on downregulation of *CD226* (**i**), cytotoxicity (**i**) and production of IFN-γ and IL-10 (**j**) in NK92 cells. NK92 cells were transfected with control mimics/inhibitors or miR-150 mimics/inhibitors (100 nM) and then treated with normoxic or hypoxic CL1-5-derived EVs for additional 5 days. The expression of cytokines and surface markers was measured by a Luminex system and flow cytometry. All data represent median values of triplicate samples and are representative of 3 independent experiments. Error bars represent SDs. * *p* < 0.05, ** *p* < 0.01, *** *p* < 0.005.

**Figure A2:**
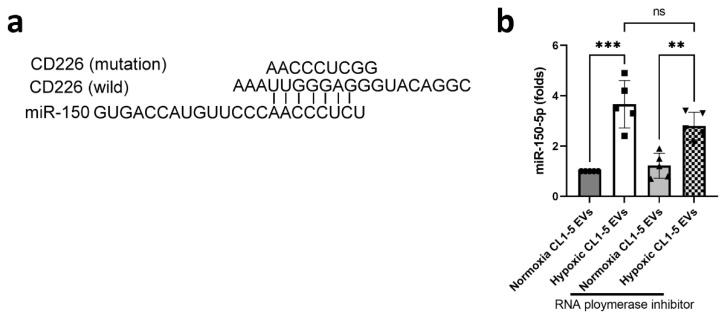
(**a**) The binding sequence for miR-150-5p in the 3′UTR of *CD226* mRNA. The miRNA target algorithms predicted that the 3′-UTR of *CD226* (wild type) had a binding site for miR-150-5p; the 3′-UTR of mutated *CD226* was designed as shown above. (**b**) RNA polymerase inhibitor did not affect the increased expression of miR-150-5p in NK cells treated with hypoxic CL1-5-derived EVs. All data represent median values of triplicate samples and are representative of 3 independent experiments. Error bars represent the SD. ** *p*  <  0.01; *** *p*  <  0.005.

To investigate how hypoxic CL1-5-derived EVs modulate the expression of CD226 in NK cells, we profiled the miRNAs by RNA sequencing in hypoxic and normoxic CL1-5-derived EVs. We found 30 markedly upregulated miRNAs in hypoxic CL1-5-derived EVs, compared with normoxic CL1-5-derived EVs (Table 1). We then used Targetscan, a predicting website, to find out the miRNAs with potential to regulate *CD226* mRNA expression. As shown in Figure A2a in Appendix A, miR-150-5p can bind to the 3′UTR of *CD226* mRNA and luciferase reporter analysis also supported the interaction between miR-150-5p and *CD226* (Figure 2a). qRT-PCR analysis revealed that hypoxia stimulated CL-1-5 cells to produce miR-150-5p-enriched EVs (Figure 2b), and that the level of miR-150-5p in both primary NK and NK92 cells was elevated after treatment with hypoxic CL1-5-derived EVs (Figure 2c). Treatment with CL1-5-derived EVs did not significantly alter the viability of primary NK and NK92 cells (Figure 2d) or the expression of pri-/pre-miR-150 in NK92 cells (Figure 2e). RNA polymerase inhibitor failed to prevent an increase in the levels of miR-150-5p, indicating that the miR-150-5p was exogenous (obtained from EVs) rather than endogenous in NK cells (Figure A2b in the Appendix A). In addition, NK92 cells transfected with miR-150-5p mimics had decreased *CD226* expression, lytic ability and IFN-γ production, as well as increased IL-10 expression (Figure 2f–h). Conversely, miR-150-5p inhibitors ameliorated the effects of hypoxic CL1-5 derived EVs on downregulation of *CD226*, reduced cytotoxicity, reduced IFN-γ production and increased IL-10 secretion in NK92 cells (Figure 2i,j).

### 3.3. Hypoxic Cancer-Derived NK Cells Show Enhanced Inflammation and Production of Pro-Tumorigenic Factors

**Figure 3:**
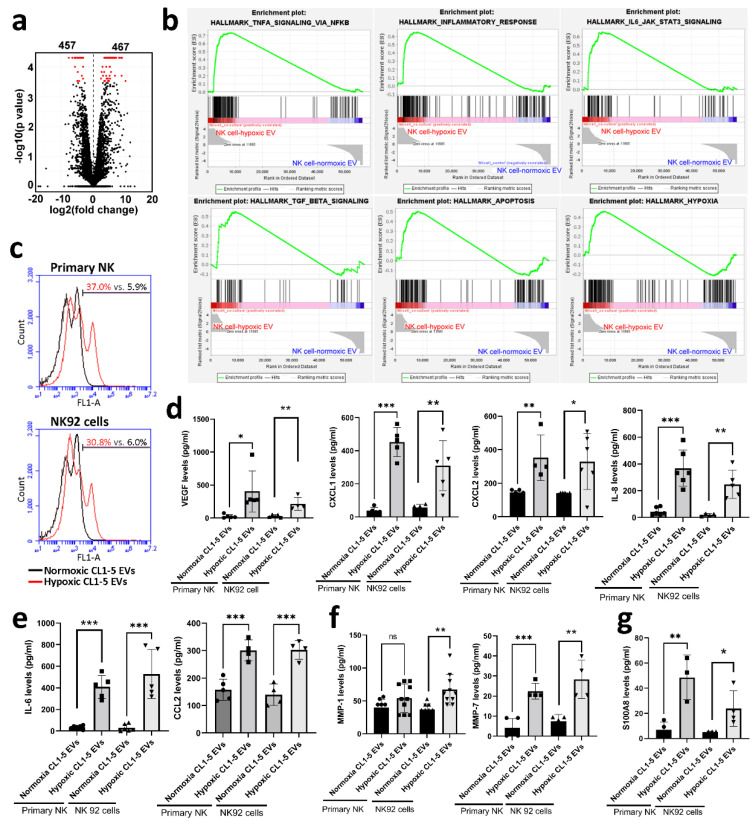
The transcriptome and secretome of hypoxic CL1-5-derived EV-treated NK cells. (**a**) The mRNA profiles of primary NK cells treated with hypoxic CL1-5-derived EVs. (**b**) Gene Set Enrichment Analysis (GSEA) of the transcriptomes in primary NK cells treated with hypoxic and normoxic CL1-5-derived EVs. (**c**) The cell proliferation of NK cells. The levels of pro-angiogenic factors (**d**), inflammatory factors (**e**), tissue remodeling factors (**f**) and an immune-suppressor (**g**). Primary NK cells were treated with normoxic or hypoxic CL1-5-derived EVs for 5 days, the transcriptome and secretome of NK cells were assessed with RNA-sequencing and Luminex system, respectively. For cell proliferation analysis, primary NK and NK92 cells were stained with carboxyfluorescein diacetate, succinimidyl ester (CFSE) and then treated with normoxic or hypoxic CL1-5-derived EVs for 5 days. The fluorescence intensity of CFSE was measured by flow cytometry. All data represent median values of triplicate samples and are representative of 3 independent experiments. Error bars represent SDs. * *p* < 0.05, ** *p* < 0.01, *** *p* < 0.005. Abbreviation: EV, extracellular vesicle.

**Figure A3:**
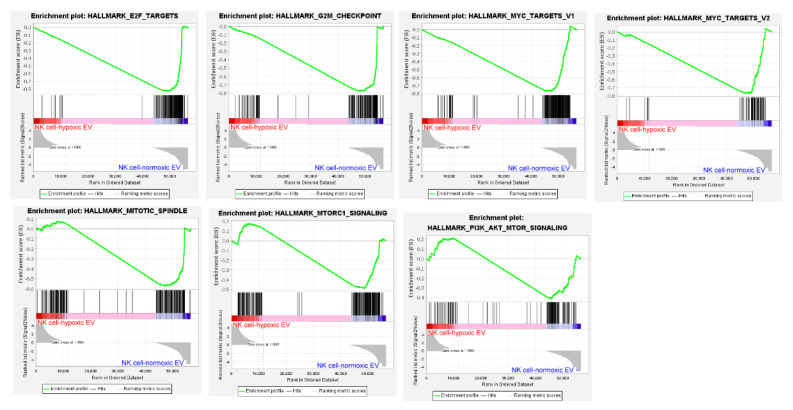
Gene Set Enrichment Analysis (GSEA) of the transcriptomes in primary NK cells treated with hypoxic and normoxic CL1-5-derived EVs.

To investigate the acquisition of phenotypic alterations in hypoxic cancer-derived NK cells, we assessed the transcriptomes of NK cells with NGS. There were 924 differentially expressed genes (with the log_2_(fold-change) >1 and *p*-value < 0.05), including 467 up- and 457 down-regulated genes, in primary NK cells treated with hypoxic lung cancer-derived EVs (Figure 3a). Gene Set Enrichment Analysis (GSEA) revealed several inflammatory pathways in NK cells, which were significantly affected by hypoxic CL1-5-derived EVs, including TNFA_SIGNALING_VIA_NFKB, INFLAMMATORY_RESPONSE, IL6_JAK_STAT3_SIGNALING, TGF_beta_SIGNALING, HYPOXIA and APOPTOSI (Figure 3b). In contrast, cell proliferation-related pathways, including E2F_TARGETS, MYC_TARGETS, MITOTIC_SPINDLE and MTORC1_SIGNALINGs, were significantly influenced by normoxic CL1-5-derived EVs (Figure A3 in Appendix A). Further analysis showed that hypoxic CL1-5-derived EVs appeared to decrease the proliferation of primary NK and NK92 cells (Figure 3c). These findings suggest that hypoxic lung cancer-derived EVs might reduce the anti-cancer activity of NK cells by decreasing mitogen signaling, which in turn inhibits NK cell proliferation.

Since an inflammatory response was triggered in NK cells, we characterized the secretome from NK cells treated with hypoxic lung cancer-derived EVs. Transcriptome analysis revealed that 24 genes of secretory factors were upregulated in NK cells treated with hypoxic CL1-5-derived EVs (Table 2). The overall secretome analysis revealed signatures characterizing hypoxic lung cancer EV-educated primary NK and NK92 cells involved inflammation and angiogenesis (VEGF, CXCL1, CXCL2 and IL-8/CXCL8), inflammatory chemokines (IL-6 and CCL2), tissue remodeling (MMP-1 and MMP-7) and immuno-suppressive factors (S100A8) at the protein level (Figure 3d–g).

**Figure 4:**
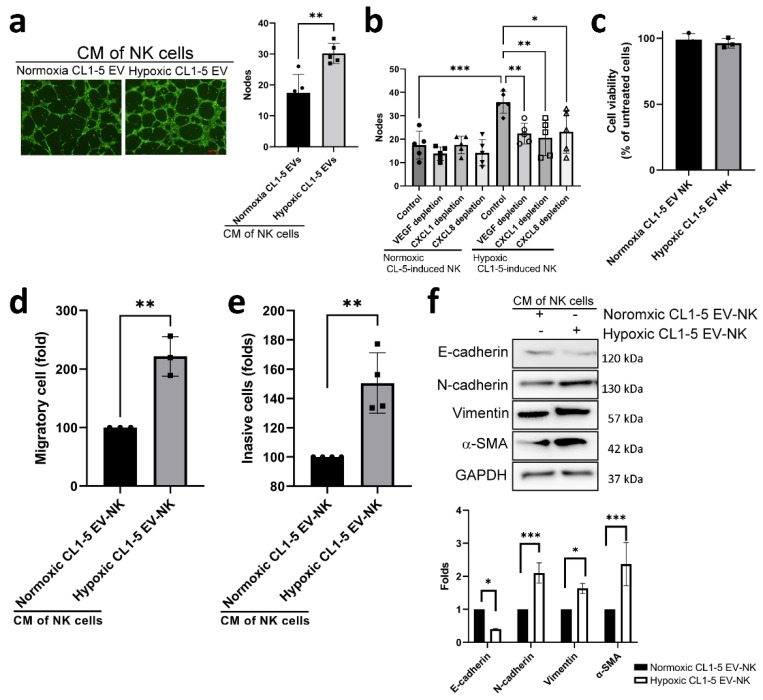
Hypoxic CL1-5-derived EV-treated NK cells promoted angiogenesis and cancer progression. (**a**) The condition medium (CM) of NK cells treated with hypoxic lung cancer-derived EVs increased tube formation of HUVECs, showing increased node counts. (**b**) Various soluble factors, including VEGF, CXCL1 and CXCL8, involved in the angiogenesis, are shown by increased tube formation of HUVECs, promoted by CM released from NK cells treated with hypoxic CL1-5 cells. The CM of NK cells treated with hypoxic lung cancer-derived EVs did not alter the viability of CL1-5 cells (**c**) but could increase migration (**d**), invasion (**e**) and epithelial-mesenchymal transition (EMT) (**f**) of CL1-5 cells. NK92 cells were treated with normoxic or hypoxic CL1-5-derived EVs for 5 days. After washing, the CMs of NK cells were collected after a 48-h incubation. CM of NK cells was added to the HUVEC on Matrigel coated 48-well plates and the degree of tube formation of HUVEC was captured by a fluorescence microscope after an 8-h incubation. For migration and invasion analysis, CL1-5 cells were seeded in 8-μm pore inserts, and CM of NK cells was added into the button well, and the migration of cancer cells was assessed by crystal violet staining after the indicated times (24 h for migration and 48 h for invasion). CL1-5 cells were treated with CM of NK cells for 24 h, and the EMT markers were assessed by Western blot analysis after a 24-h incubation. All data represent median values of triplicate samples and are representative of 3 independent experiments. Error bars represent SDs. * *p* < 0.05, ** *p* < 0.01, *** *p* < 0.005. Abbreviation: EV, extracellular vesicle.

Tube formation analysis also showed that the conditioned medium (CM) of NK cells treated with hypoxic lung cancer-derived EVs exhibited pro-angiogenic potential (Figure 4a). Depletion of angiogenic factors, including VEGF, CXCL1 and IL-8/CXCL8, prevented tube formation mediated by these NK cells educated by the hypoxic cancer-derived EVs, showing that they all contributed to tumor-associated angiogenesis (Figure 4b). The CM of NK cells treated with hypoxic lung cancer-derived EVs did not alter the viability of CL1-5 cells (Figure 4c) but could increase lung cancer migration and invasion, as well as epithelial–mesenchymal transition (EMT) in CL1-5 cells (Figure 4d,f).

### 3.4. miR-150 Inhibitor Improved Lung Cancer Growth in a Mouse Model

**Figure 5:**
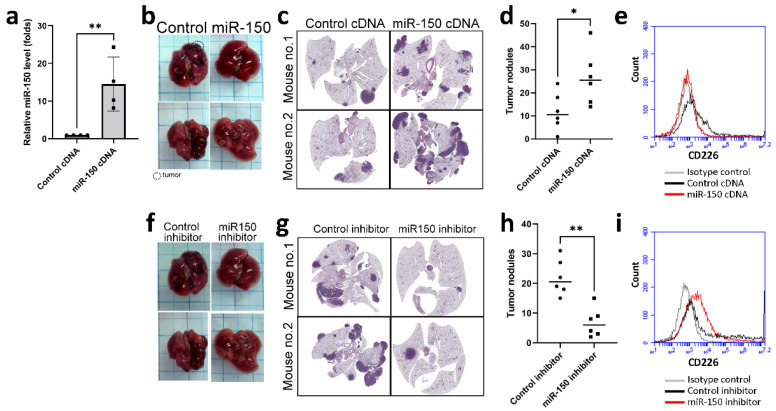
miR-150-5p could be a target for lung cancer treatment in vivo. (**a**) Overexpression of miR-150-5p. LLC cells were transfected with either control or miR-150-5p cDNA plasmid, and the stable clone was established by G418 selection. Data are presented in mean ± SD of four replicates. (**b**–**e**) Overexpression of miR-150-5p increased the number of tumor nodules in the lungs of mice in an allograft model, as determined by gross examination (**b**), microscopy with H&E staining (**c**) and counting of the tumor nodules (**d**). (**e**) The level of CD226 in NK cells isolated from the lungs of miR-150-5p-overexpressing LLC-bearing mice. (**f**–**i**) The inhibitors of miR-150-5p amend the growth of LLC in the lungs of mice, as determined by gross examination (**f**), microscopy with H&E staining (**g**) and counting of the tumor nodules (**h**). (**i**) The expression of CD226 in NK cells isolated from the lungs of miR-150-5p-overexpressing LLC-bearing mice treated with either control or miR-150 inhibitor. (**d**,**h**) The number of tumor nodules in each mouse is plotted and the median value of each group is presented. * *p* < 0.05, ** *p* < 0.01.

To assess the biologic role of miR-150-5p in vivo, we engineered LLC cells that overexpressed miR-150-5p and then implanted them into mice via tail vein injection. Overexpression of miR-150-5p increased the development of tumor nodules in the lungs of mice (Figure 5a–d). Consistent with the previous data, there was decreased expression of CD226 in NK cells isolated from the lungs of mice with miR-150-overexpressing LLC tumors, compared to those from mice implanted with wild-type LLC (Figure 5e).

To evaluate whether miR-150-5p could be a therapeutic target for lung cancer, LLC-bearing mice were treated with either a control inhibitor or miR-150-5p inhibitor. The inhibition of miR-150-5p decreased the development of lung cancer in vivo (Figure 5f–h). The expression of CD226 on NK cells isolated from mice that received miR-150-5p inhibitor, was also reversed (Figure 5i).

### 3.5. Elevated miR-150-5p Was Observed in Lung Cancer Patients

**Figure 6:**
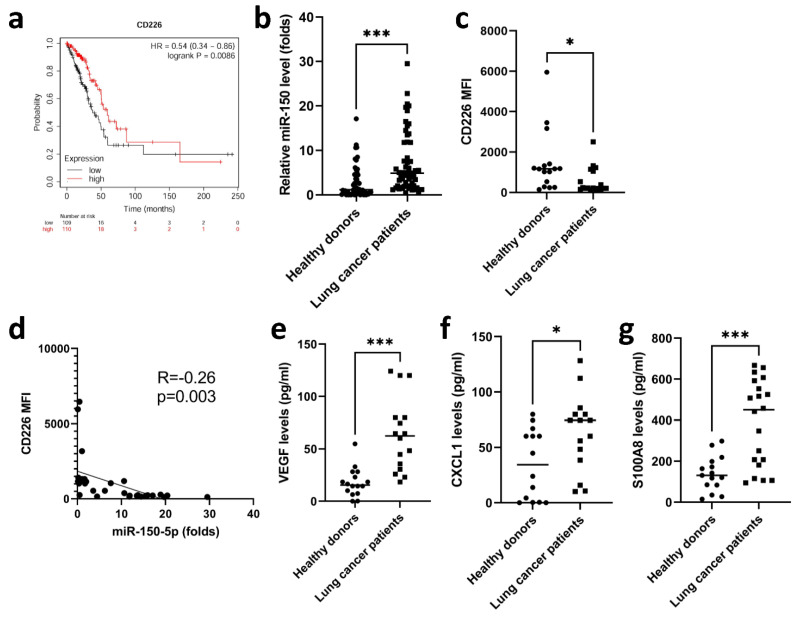
(**a**) Analysis of data from patients with lung cancer, obtained from TCGA. Overall survival of patients grouped according to higher or lower expression (compared with the median) of *CD226* (*n*  =  110 (high) and *n*  =  109 (low)). (**b**) Significantly higher level of miR-150-5p in EVs isolated from the serum of patients with lung cancer than in those from health donors. (**c**) EVs isolated from lung cancer patients decreased the expression of CD226 in NK92 cells. (**d**) The correlation of miR-150-5p with the effect of CD226 downregulation. Upregulation of VEGF (**e**), CXCL1 (**f**) and S100A8 (**g**) were noted in NK92 cells treated with the EVs isolated from lung cancer patients. The expression of cytokines and surface markers was measured by a Luminex system and flow cytometry. All data represent median values of triplicate samples and are representative of 3 independent experiments. Error bars represent SEMs. * *p* < 0.05, *** *p* < 0.005. Abbreviation: MFI, mean fluorescence intensity.

To establish whether there is an association between CD226 expression on NK cells and overall survival in patients with lung cancer, we analyzed public data from The Cancer Genome Atlas (TCGA). The dataset showed that CD226^low^ NK cell infiltration was associated with poorer overall survival in patients with lung cancer (Figure 6a). We also assessed the levels of miR-150-5p in lung cancer patients, showing that miR-150-5p was elevated in the serum of lung cancer patients, compared with healthy donors (Figure 6b). Treatment of NK92 cells with EVs isolated from the serum of lung cancer patients, decreased their expression of CD226 (Figure 6c), and this effect was significantly, yet weakly, correlated with the level of miR-150-5p in the serum EVs (R = −0.26, *p* = 0.003) (Figure 6d). In addition, the EVs isolated from the serum of lung cancer patients also stimulated NK cells to produce VEGF, CXCL1 and S100A8, which contributed to angiogenesis and immune suppression in the TME (Figure 6e–g).

## 4. Discussion

**Figure 7:**
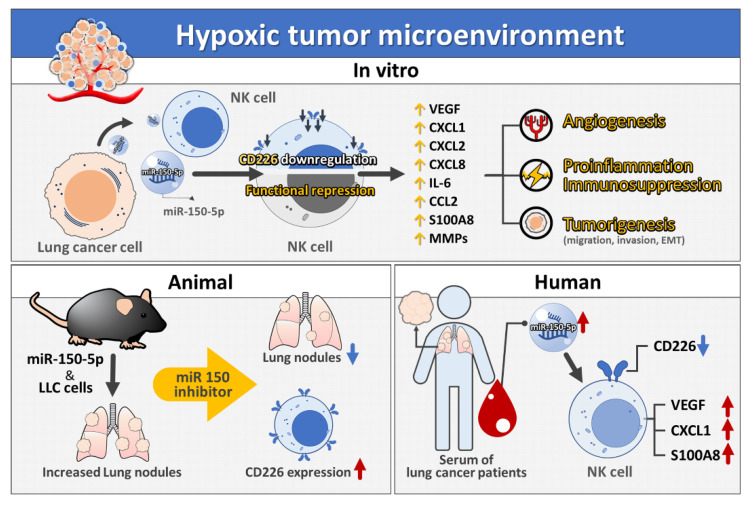
Summary of the study result. Hypoxic lung cancer-derived miR-150-5p-rich EVs downregulated CD226 expression of NK cells, which exhibited a pro-inflammatory, pro-angiogenic and tumorigenesis phenotype.

Lung adenocarcinoma is the most prevalent type of lung cancer. The one-year survival rate in people diagnosed with advanced lung cancer is typically <20%, and lung cancer constitutes one of the major challenges in biomedical and clinical research. Most studies on the mechanisms of immune evasion in oncology have focused on T cell immune responses against tumor tissue. In contrast, we investigated the pathogenic alteration of the phenotype and function of NK cells in the current study. We phenotypically and functionally characterized NK cells after hypoxic lung cancer priming and then investigated their roles in promoting tumorigenesis, immune dysregulation and angiogenesis (Figure 7). Hypoxia-stimulated lung cancer negatively regulated NK cell-dependent anti-tumor immune responses by EV transfer. Therapeutic targeting of EV biomaterial was shown to activate NK cells and promote anti-tumor immunity via enhancing the suppression of cancer by NK cells in mice.

NK cells have been found to be compromised in several cancer types, and skewed NK cells contributes to cancer progression via tumor escape and immunosuppression. Downregulation of activating receptor expression on NK cells and impaired NK function are common mechanisms of immune evasion in cancer, and have been associated with tumor progression [28]. Competitive interactions between TIGIT (T cell immunoreceptor with Ig and immunoreceptor tyrosine-based inhibitory motif [ITIM] domains) and CD226 receptors with cognate ligands, such as nectins and poliovirus receptor (PVR), play a critical role in TIGIT-driven immune suppression, and anti-TIGIT therapy is designed to reverse this suppression by inhibiting TIGIT-PVR binding [29]. A recent study reported that the effectiveness of TIGIT blockade depends on CD226 activation, and that immune cells with low levels of CD226 did not respond to antigen stimulation or anti-TIGIT monoclonal treatment [30]. It has been reported that CD226 was decreased in peripheral blood NK cells from patients with gastric or lung cancer [31]. It has been presumed that eomesodermin-dependent transcriptional mechanisms and a PVR-mediated ubiquitin–proteasome degradation are involved in the expression of CD226 in T cells [32,33], whereas the mode of CD226 expression in NK cells has not yet been fully elucidated. In the current study, we found that hypoxia stimulated lung cancer cells to produce EVs, which deliver miR-150-5p to NK cells and then polarize them causing dysfunction. miR-150-5p in the EVs not only decreased the cytotoxicity of NK cells but it also reduced cell proliferation in NK cells by decreasing CD226 expression. In addition, in vivo blockade of miR-150-5p decreased lung cancer growth through improvements in NK cell function in mice. Importantly, circulating miR-150-5p were increased in the serum of patients with lung cancer, and these increased serum levels were positively associated with the ability to decrease the expression of CD226 in NK cells. Understanding these mechanisms and their relevance to hypoxia and NK cell polarization may offer further incentives for targeting miR-150-5p as a promising strategy for next-generation cancer immunotherapy.

The crosstalk between NK cells and the TME plays a crucial role in protection against cancer development, cancer outcomes and immunotherapy responses [34]. Tumor angiogenesis is a complex and multi-stage process, which is usually induced by hypoxia, and angiogenesis factors drive immune suppression by directly suppressing antigen-presenting cells as well as immune effectors, including M2-tumor-associated macrophages, regulatory T cells and myeloid-derived suppressor cells; this also drives tumor angiogenesis, creating a vicious cycle between immune and endothelial cells [35]. Decidual NK (dNK) cells are a unique NK cell subset at the main maternal-fetal interface, which have been indicated to participate in vascularization during embryonic and placental development [36]. Cells with a phenotype similar to dNK (CD56^bright^CD16^low^CD49^+^CD9^+^ decidual-like NK) cells and the nurturing activity of NK cells have been described in cancer, indicating the relevance of NK cells in the development of tumor angiogenesis [37,38]. CD56^bright^CD16^−^ cells have also been identified as the predominant tumor subset of NK cells in lung cancer [39]. The major factors contributing to peripheral blood NK-cell phenotype remodeling are TGF-β, glycodelin-A (GdA), prostaglandin E2 (PGE2), and hypoxia [40]. Our results showed that hypoxic lung cancer-derived EVs increased the polarization of NK cells to dNK-like cells with pro-angiogenic phenotypes, which have the ability to support angiogenesis in vitro with several soluble factors including, VEGF, CXCL1/2 and IL-8. There is a need for extensive research concerning the potential triggers that can contribute to the proangiogenic properties of NK cells, so we can deepen our understanding of the pathogenic mechanisms and develop more effective anti-VEGF therapies and immunotherapies.

Growing evidence suggests that the TME supports many tumor-sustaining processes, such as cell proliferation, tumor growth and metastasis, and that several factors are involved in the architecture of the TME [41,42]. Signaling of CXCR2 and its ligands (CXCL8 and CXCL1) leads to the recruitment of tumor-promoting immune cells, such as tumor-associated macrophages and MDSCs [43,44]. S100A8/A9 has been described as an important factor for the recruitment of MDSCs and the stimulation of their immunosuppressive functions in the TME, but it also plays a critical role in the formation of the pre-metastatic niche within organs that these cells metastasize to [45,46,47]. In our study, we showed for the first time that NK cells stimulated with hypoxic lung cancer-derived EVs undergo tumor immune microenvironment editing, which is characterized by the up-regulation of pro-tumorigenesis cytokines (IL-6 and CCL2) and pro-angiogenic chemokines (VEGF, IL-8 and CXCL1) and those with immunosuppressive features (IL-10 and S100A8), as well as factors involved in the ECM remodeling cascade (MMP-1, MMP-7, MMP-14 and MMP-17). EVs isolated from lung cancer patients also stimulated NK cells to produce higher levels of these cytokine/chemokines compared with those from healthy donors, introducing a new scenario for possible remodeling activities of the TME by NK cells in lung cancer.

The role of miR-150 in cancer has been investigated in many studies, yet the results appear heterogenous. Patients of NSCLC with higher plasma level of miR-150 had worse disease-free survival than those with lower level [48]. Upregulation of miR-150-5p was observed in NSCLC tissue and cell lines; miR-150-5p promoted proliferation and migration of lung cancer cells, as well as suppressed their apoptosis [49]. The serum level of miR-150-5p was significantly higher in patients of renal cell carcinoma than healthy control subjects [50]. However, many other studies reported the tumor suppressive effects of miR-150-5p. Significantly lower expression of miR-150-5p was observed in cancer stem cells of non-small cell lung cancer, which was associated with poorer outcome; miR-150-5p inhibited sphere formation and metastatic colonization of cancer stem cells [51]. Similarly, miR-150 inhibited liver cancer stem cells [52]. The expression of miR-150-5p was significantly lower in hepatocellular carcinoma tissues, particularly in metastatic cancer tissues, suggesting the potential effects of miR-150-5p inhibition in promoting cancer migration and invasion [53]. Lower expression of miR-150-5p in colorectal cancer tissues was associated with poorer overall survival; miR-150-5p inhibited proliferation, migration, and invasion of colorectal cancer cells as well as angiogenesis [54]. The serum exosomal miR-150-5p level was significantly lower in colorectal cancer patients than healthy subjects [55]. In melanoma cells, miR-150-5p sponged circular RNA VANGL1, which might promote the proliferation, migration and invasion of melanoma cells [56].

Our study has two major limitations. Firstly, EVs used in this study were isolated using exosome isolation kits and characterized by using transmission electron microscopy, immunoblot (with exosome markers, such as CD9, CD63, CD81 and HSP70), and total protein quantification as in our previous studies [16,17,18]. Although these EVs presented exosome markers and showed exosome characteristics, we were not able to confirm if they were exosomes. We therefore use the term “EVs” in the current report. Indeed, a variety of isolation methods based on different principles, such as ultracentrifugation, density gradient, precipitation, immune precipitation, membrane affinity, size-exclusion chromatography, iodixanol gradient and phosphatidylserine affinity, are available [40,57]. Different isolation methods yielded EVs with different profiles in terms of morphology, particle size, and content. Although no perfect and standardized method is available currently, the research field of EVs is still promising and deserves further studies. Secondly, we used only EVs derived from CL1-5 cells, a highly invasive lung cancer cell line. In our previous studies about the role of EVs derived from hypoxic lung cancer cells, we have used several lung cancer cell lines (such as CL1-5, NCI-H2087, NCI-H1792 and NCI-H1437) [16,17]. EVs isolated from these cell lines in hypoxic condition presented similar characteristic and functional changes, compared with those isolated from cells in normoxic condition. We therefore believe that EVs from other lung cancer cell lines present similar functional characteristics as those from CL1-5 cells. Nevertheless, further study is needed to confirm our findings in other cancer cell lines.

## 5. Conclusions

The present study reports a previously undescribed immuno-modulatory effect of miR-150-5p in hypoxic lung cancer-secreted EVs, which can reprogram NK cells to a dysfunctional pro-inflammatory and pro-angiogenic phenotype. In the broad concept of immune regulation beyond tumor immunity, we have delineated a previously undescribed mechanism related to skewed NK cell function plasticity in the TME. Our study highlights the potential for targeting miR-150 to improve NK cell-based cancer immunotherapy.

## Figures and Tables

**Table 1:** The upregulated miRNAs in extracellular vesicles from lung cancer cells cultured in hypoxic condition.

miRNA	Fold Change	miRNA	Fold Change	miRNA	Fold Change
hsa-miR-10b	2.39	hsa-miR-122-5p	2.67	hsa-miR-195	2.39
hsa-miR-3200	2.36	hsa-miR-1306	2.69	hsa-miR-200c	2.21
hsa-miR-320d	2.12	hsa-miR-103	2.24	hsa-miR-26b	2.01
hsa-miR-23a	5.91	**hsa-miR-150**	**2.30**	hsa-miR-3074	2.95
hsa-miR-34b	3.38	hsa-miR-3648	5.78	hsa-miR-4467	2.72
hsa-miR-3615	3.19	hsa-miR-3687	3.16	hsa-miR-4488	5.54
hsa-miR-4532	3.01	hsa-miR-3934	2.54	hsa-miR-4492	6.46
hsa-miR-4664	3.43	hsa-miR-3960	2.64	hsa-miR-451a	2.26
hsa-miR-501	5.08	hsa-miR-619	5.07	hsa-miR-769	2.09
hsa-miR-549a	3.49	hsa-miR-760	2.22	hsa-miR-7977	7.37

**Table 2:** The upregulated genes of secretory factors in NK cells treated with hypoxic CL1-5-derived extracellular vesicles.

Category	Gene	Folds
Angiogenesis	*EDN1*	98.41
	*VEGFA*	15.01
	*CXCL2*	146.34
	*CXCL3*	122.62
	*CXCL8*	28.50
	*CXCL1*	27.58
Extracellular matrix degradation	*MMP1*	68.46
	*MMP17*	70631.4
	*MMP14*	24.91
	*MMP7*	10.57
Immuno-suppression	*S100A8*	9.59
	*S100A9*	11.51
Inflammation	*IL1B*	188.97
	*IL6*	89.15
	*IL1A*	81.40
	*IL12B*	62.42
	*IL23A*	31.39
	*IL27*	16.25
	*IL10*	9.54
	*CXCL5*	40.95
	*CCL8*	20.97
	*CXCL16*	9.43
	*CCL2*	9.33
	*CCL7*	7.90

## Data Availability

Data are available from the authors upon reasonable request.
